# Transcriptomic and Physiological Analyses of Two Rice Restorer Lines under Different Nitrogen Supplies Provide Novel Insights into Hybrid Rice Breeding

**DOI:** 10.3390/plants12122276

**Published:** 2023-06-11

**Authors:** Xiaojian Qin, Xiaowei Li, Juan Xiao, Qian Wu, Yuntong Li, Cuiping Li, Dan Jiang, Tingting Tang, Wenbin Nan, Yongshu Liang, Hanma Zhang

**Affiliations:** 1 College of Life Sciences, Chongqing Normal University, Chongqing 401331, China; m17727268158@163.com (X.L.); 15310319465@163.com (J.X.); wq1968619@163.com (Q.W.); lh15213222686@126.com (Y.L.); lcp132103007@126.com (C.L.); njsgskl@163.com (D.J.); tingtingtang_15@163.com (T.T.); nanwenbin513@163.com (W.N.); yongshuliang@yeah.net (Y.L.); hanmazhang@126.com (H.Z.); 2 Key Laboratory of Molecular Biology of Plants Environmental Adaptations, Chongqing Normal University, Chongqing 401331, China

**Keywords:** *Oryza sativa*, restorer lines, physiology, nitrogen-use efficiency, transcriptome

## Abstract

Improving plant nitrogen-use efficiency (NUE) has great significance for various crops, particularly in hybrid breeding. Reducing nitrogen inputs is key to achieving sustainable rice production and mitigating environmental problems. In this study, we analyzed the transcriptomic and physiological changes in two indica restorer lines (Nanhui511 [NH511] and Minghui23 [MH23]) under high nitrogen (HN) and low nitrogen (LN) conditions. Compared to MH23, NH511 was more sensitive to different nitrogen supplies and exhibited higher nitrogen uptake and NUE under HN conditions by increasing lateral root and tiller numbers in the seedling and maturation stages, respectively. NH511 also exhibited a lower survival rate than MH23 when planted in a chlorate-containing hydroponic solution, indicating its HN uptake ability under different nitrogen-supply conditions. Transcriptomic analysis showed that NH511 has 2456 differentially expressed genes, whereas MH23 had only 266. Furthermore, these genes related to nitrogen utilization showed differential expression in NH511 under HN conditions, while the opposite was observed in MH23. Our findings revealed that NH511 could be regarded as elite rice and used for breeding high-NUE restorer lines by regulating and integrating nitrogen-utilization genes, which provides novel insights for the cultivation of high-NUE hybrid rice.

## 1. Introduction

Rice (*Oryza sativa* L.) is a staple crop that feeds over half of the global population. The global food crisis has become a critical issue and is predicted to worsen over the next 20 years owing to the increasing global population [1,2]. Hybrid breeding is one effective strategy proposed to manage the global food crisis. Over the past several decades, successful hybrid rice-breeding programs have produced rice varieties with the potential to yield ~20% more than the inbred varieties in field production [3]. Two production systems are used in hybrid rice breeding, the two-line system and the three-line system [4,5]. Elite restorers play a crucial role in hybrid breeding by benefiting the F1 generation. The identification and development of exceptional restorer cultivars are pivotal for successful hybrid seed production, directly impacting the final grain yield [6,7]. 

Nitrogen (N) is an essential macronutrient in plant growth and development that strongly influences crop productivity. N-use efficiency (NUE) is the foundation of rice production with high yield and quality, and improving NUE is an important issue in modern rice breeding [8,9]. To improve grain yield, the application of synthetic N fertilizers has increased in recent decades; however, the utilization of the applied N by crops is lower than 50% because of low N-use efficiency (NUE) [10,11,12]. Therefore, it is an effective way to identify key quantitative trait locus (QTLs) related to nitrogen-use efficiency based on the significant genetic differences among rice germplasm in recent years [13]. Multiple QTLs related to nitrogen utilization have been identified by constructing different genetic populations in rice [14,15,16]. A natural variation in rice *NRT1.1B* was identified and contributed to NUE variation between *indica* and *japonica* varieties [17]. Moreover, a variation of the *OsTCP19* promoter was identified by genome-wide association analysis (GWAS), which regulated NUE by regulating the expression of nitrogen-utilization pathway genes [18], rice *OsNPF6.1* [19], *OsLHT1* [20], and *OsARE* [21], which were also identified to regulate the nitrogen utilization based on the significant genetic variations.

Ensuring food security and promoting sustainable agricultural development through the implementation of resource-saving and environment-friendly methods have become strategic concerns over the past decades [22]. Notably, “Green Super Rice” (GSR) was proposed for rice breeding and production based on rice functional genomics research [22,23]. Many rice cultivars with different green traits have been identified and developed, providing a solid foundation for modern rice breeding and production. Improving crop NUE while maintaining crop productivity has several economic and environmental benefits [24].

Genome-wide expression analysis is a powerful approach for analyzing complex traits, such as NUE. RNA sequencing (RNA-seq), a high-throughput sequencing technology, is widely utilized in rice studies, as high-quality and suitable genome information is available for rice [25,26]. Many studies have recently investigated the rice transcriptome under various N conditions using large-scale datasets to identify differential genes and pathways that respond to N availability [27,28,29,30]. However, these studies were mostly limited to some transgenic lines with key genes identified in previous studies and focused on ordinary rice materials, which did not provide an accurate description of transcriptome-wide responses to the changes in N availability. Our previous study carried out screening and primary identification of different rice cultivar resources in China; the results showed that different rice cultivars exhibited various characteristics and responded to nitrate with NO_3_^−^ as an N source in hydroponic conditions [31]. Among these cultivars, Nanhui511 (NH511) was characterized as a sensitive cultivar, and Minghui23 (MH23) as an insensitive cultivar under different N supplies [31]. In the present study, we further investigated the physiological responses of these two rice cultivars, NH511 and MH23, under varying N-supply conditions. Moreover, we combined multiple RNA-seq analyses to assess their transcriptomic variations, focusing on two indica restorer cultivars exhibiting responses to high (HN) and low N (LN) supplies. Our combined physiological and transcriptomic analysis of rice restorers revealed their N-responsive characteristics, improved our understanding of the regulatory activities involved in the N response, and provided novel insights into breeding for high NUE using elite restorers in rice.

## 2. Materials and Methods

### 2.1. Rice Materials and Growth Conditions

The seeds of two rice cultivars (*Oryza sativa* cv. NH511 and MH23) were germinated in Murashige and Skoog (MS) media and then transferred to tap water before being transferred into a hydroponic solution (1.8 mM KCl, 0.36 mM CaCl_2_, 0.54 mM MgSO_4_, 0.32 mM KH_2_PO_4_, 40 μM Fe (II)-EDTA, 18.8 μM H_3_BO_3_, 13.4 μM MnCl_2_, 0.32 μM CuSO_4_, 0.3 μM ZnSO_4_, 0.03 μM Na_2_MoO_4_, and 1.6 mM Na_2_SiO_3_) supplied with KNO_3_ as the only N source. To fully understand the distinct growth responses of NH511 and MH23 to varying N supplies, we first germinated them and cultivated them under LN conditions for seven days. The seedlings were then divided into two groups and transferred to HN and LN conditions for six days of cultivation. The seedlings were cultivated in a growth chamber under a photoperiod of 12 h/12 h (light/dark) (~230 μmol m^−2^ s^−1^) at 28 °C/25 °C, and the solution was renewed every 3 days. For the LN and HN treatment assays, the seedlings were transferred to LN (0.2 mM KNO_3_) and HN solutions (5 mM KNO_3_), respectively. For NUE analysis, the seedlings were transferred to non-N fertilizer (0 mM KNO_3_) for LN treatment and N fertilizer (5 mM KNO_3_) for HN treatment.

### 2.2. RNA Isolation and Transcriptomic Analysis

Total cellular RNA was isolated from the samples treated for 3 h and grown under HN and LN conditions using the TRIzol method (Invitrogen, Carlsbad, CA, USA), with three biological replications used for this assay. Subsequently, the RNA samples from multiple replications were pooled in equimolar concentrations and then submitted to Biomarker Technologies (Beijing, China) for library construction. Separate libraries for each N treatment (HN and LN) and rice variety (NH511 and MH23) were constructed using the Truseq RNA Sample prep kit (Illumina, Woodslang, Singapore), according to the manufacturer’s protocol. Thus, in total, four libraries were constructed, and each library was represented by three biological replications. The libraries were sequenced using the paired-end Illumina (HiseqTM 2500) sequencing technology. Differential expression analyses of the two conditions were performed using the DESeq R package (version 1.10.1). DESeq provides statistical routines for determining differential expression in digital gene expression data using a model based on a negative binomial distribution. The resulting *p* values were adjusted using Benjamini and Hochberg’s approach to control the false discovery rate. Genes with an adjusted *p* value of <0.05, as determined by DESeq, were designated as differentially expressed.

### 2.3. Quantitative RT-PCR

First, 5 μg of RNA was isolated and analyzed. After checking the quality and spectrophotometric quantification, 2 μg RNA was converted into cDNA using M-MLV Reverse Transcriptase (Invitrogen, USA) following the manufacturer’s protocol. Each sample was analyzed in triplicate using at least two cDNA preparations by qRT-PCR, and TaKaRa SYBR Pre-mix Ex-TaqII reagent kits were used for real-time PCR analysis. The data were obtained by the Roche Light Cycler 480 system (Roche, Zurich, Switzerland). The specificity of the reactions was verified by carrying out a melting (dissociation) curve analysis. The expression variation in the N-related genes NH511 and MH23 was calculated, and *ACTIN1* was used as an endogenous control. The primers used in this experiment are listed in Supplementary Table S1.

### 2.4. Enzyme Activity, N Content, and NUE Analysis

Fifteen-day-old hydroponically grown seedlings were used for the nitrate reductase (NR) and glutamine synthase (GS) activity assays. The maximal in vitro activity of NR was measured according to a previously reported method [32], and an enzyme assay kit (Grace Biotechnology, Suzhou, China) was used to analyze GS activity. The total N content was measured by Grace Biotechnology, and the NUE was calculated using the following equation [33]:

NUE (%) = (TN_F_ − TN_0_)/N × 100

where TN_0_ is the total N content of plants in the non-N fertilizer treatment group; TN_F_ is the total N content of plants in the N fertilizer treatment group, and N is the total amount of N applied.

### 2.5. Chlorate Sensitivity Assay

The seedlings grown in 2 mM KNO_3_ Kimura B solution for 5 days were treated with 2 mM chlorate for 3 days and subsequently recovered in 2 mM KNO_3_ provided in Kimura B solution for 5 days. The survival rates of the control and treated seedlings were then calculated [34].

### 2.6. Field Trial of Rice Cultivars

In 2019 and 2020, NH511 and MH23 plants were grown in Chongqing (106° E, 29° N) for field tests using different N supplies. The plants were planted in 10 rows, with 30 plants per row in each plot, and three replicates were used for each N condition. Urea was used as the N fertilizer at concentrations of 80 kg N hm^−2^ for the low LN treatment and 500 kg N hm^−2^ for the HN treatment. The plants at the edge of each plot were omitted from the analyses to avoid margin effects.

### 2.7. Agronomic Trait Analyses

Agronomic traits, such as plant height, tiller numbers, 1000-grain weight, and grain yield, were measured according to a method developed in a previous study [17].

## 3. Results

### 3.1. Morphological Variations in NH511 and MH23 Grown under HN and LN Conditions

**Figure 1:**
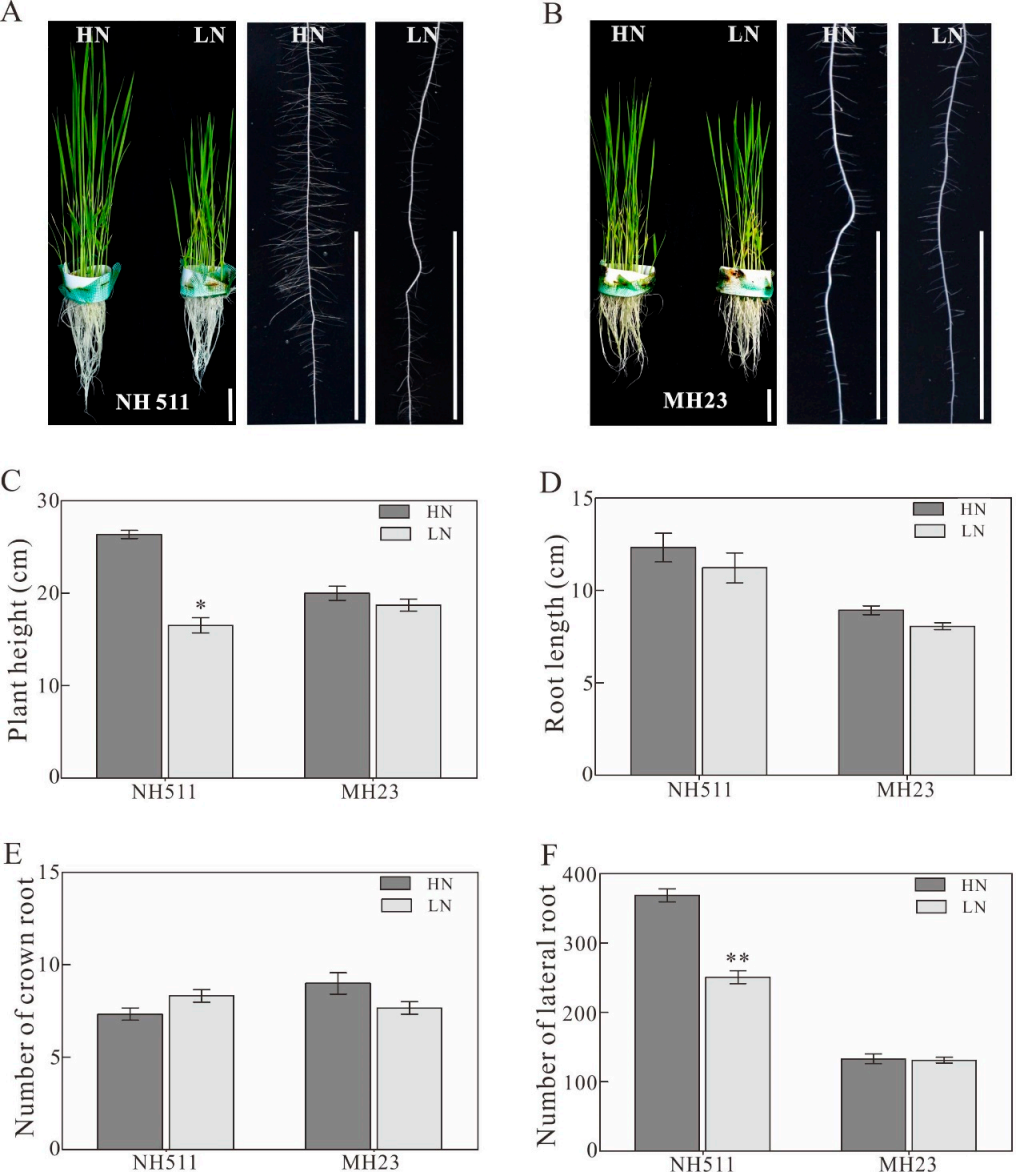
Phenotype variations in NH511 and MH23 under high nitrogen (HN) and low nitrogen (LN) conditions. (**A**) Performance of NH511 under HN and LN supplies. Scale bar = 3 cm. (**B**) Performance of MH23 under HN and LN supplies. (**C**) Plant height. (**D**) Statistical analysis of root length in NH511 and MH23. (**E**) Number of crown root analyses in NH511 and MH23. (**F**) Number of lateral roots in NH511 and MH23. The statistical analysis of comparing HN with LN was performed by *t*-tests in NH511 and MH23. **, *p* < 0.01; *, *p* < 0.05.

Our previous study showed that different rice materials exhibit different morphological and physiological variations when grown under different N supplies. In the present study, based on previous large-scale screening and preliminary results, we selected two typical rice restorer lines, NH511 and MH23, for further analysis. We found that NH511 is more sensitive to HN conditions than MH23 under different nitrogen supplies. Notably, NH511 exhibited fast growth and increased lateral root number and length under HN conditions; however, the MH23 phenotype showed no evident differences in these characteristics (Figure 1A,B). Moreover, statistical analysis revealed a significant difference in plant height and the number of lateral roots between NH511 plants treated with HN and those treated with LN (Figure 1C,F); however, no significant differences were observed in crown root number and length (Figure 1D,E). In summary, the results indicate that NH511, unlike MH23, exhibits N-responsive characteristics as a restorer, thereby promoting seedling growth through the regulation of lateral development and plant height.

### 3.2. NH511 Enhances NUE Based on Chlorate Assays

**Figure 2:**
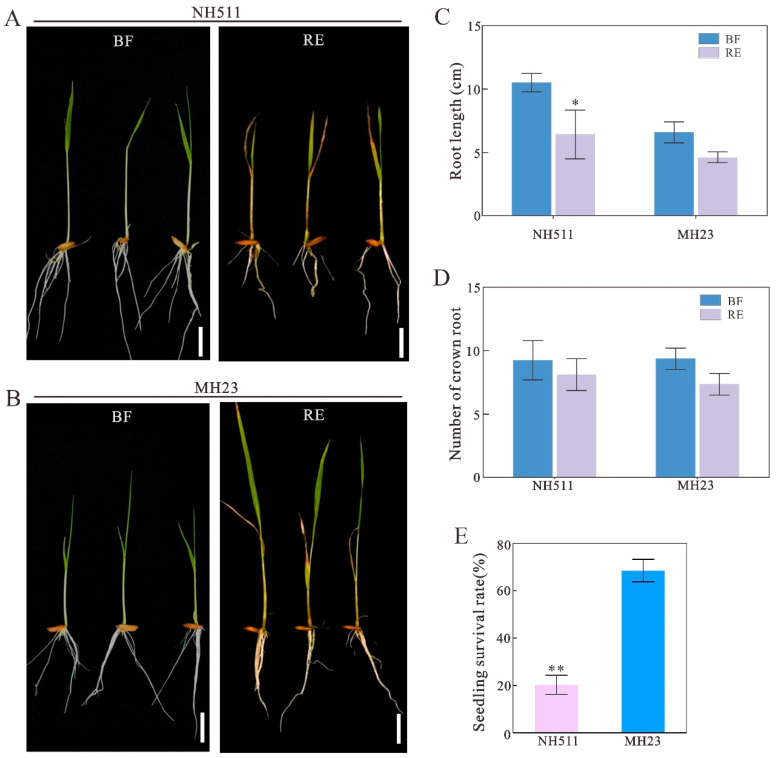
Chlorate assays for NH511 and MH23. (**A**) Phenotype of NH511 under 2 mM chlorate treatment. BF, before treatment. RE, recovery. Scale bar = 3 cm. (**B**) Phenotype of NH511 under 2 mM chlorate treatment. (**C**) Root length analysis before and after treatment. (**D**) Statistical analysis for the number of crown roots. (**E**) Statistical analysis of survival rate between NH511 and MH23. Statistical analysis comparing BF with RE was performed by *t*-tests in NH511 and MH23. **, *p* < 0.01; *, *p* < 0.05.

The observed growth response to N led us to further investigate whether NH511 exhibits more efficient modulation of nitrate uptake and assimilation than MH23 under the same conditions. To assess this, we germinated NH511 and MH23 seeds and grew them under normal N conditions for five days. Subsequently, the seedlings were exposed to a chlorate-containing hydroponic solution for the treatment assay. After transferring the plants back to normal conditions for recovery growth, we observed that NH511 displayed inhibited growth compared to MH23 (Figure 2A,B). We further analyzed the root length and found significant differences in NH511 before and after the treatment (Figure 2C); however, the number of roots remained unchanged under the same conditions (Figure 2D). Additionally, the survival assay data revealed a remarkable disparity between the two cultivars, with NH511 exhibiting a lower survival rate (~20%) than MH23 (~70%) (Figure 2E). Overall, the results from the chlorate assay indicate that NH511 possesses excellent NUE for seedling growth, contributing to our understanding of the variances among rice restorer cultivars and laying the foundation for high-NUE rice breeding.

### 3.3. Effect of Different N Supplies on N-Metabolizing Enzymes

**Figure 3:**
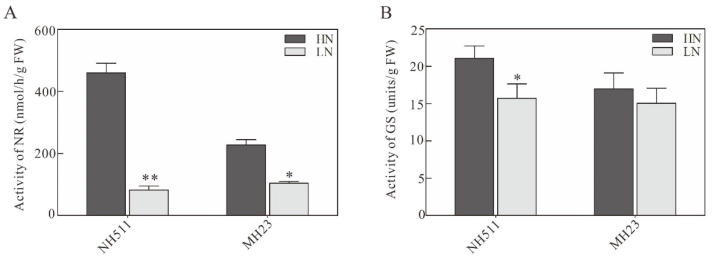
Analysis of (**A**) nitrate reductase (NR) and (**B**) glutamine synthase (GS) activities under high nitrogen (HN) and low nitrogen (LN) conditions, respectively. Statistical analysis comparing HN with LN was performed by *t*-tests in NH511 and MH23. **, *p* < 0.01; *, *p* < 0.05.

The NUE of rice depends on the activity of enzymes related to N utilization. To understand whether the activity of these enzymes increases under HN conditions in NH511, we further analyzed the activity of NR and GS in NH511 and MH23 under HN and LN conditions. The results of the enzymatic activity analysis showed that both NH511 and MH23 had low activity of NR under LN conditions, which significantly improved under HN conditions, with NH511 having a higher NR activity in the HN treatment than in the LN treatment (Figure 3A). Similarly, in NH511, GS activity also significantly increased under HN conditions compared to that under LN conditions; however, no significant change was observed in the GS activity of MH23 between the two treatments (Figure 3B). Furthermore, soluble protein content was not significantly different under different N supplies in both NH511 and MH23 (Figure S1). These results implied that NH511 also improved its N assimilation by increasing N-metabolizing enzymes under HN conditions.

### 3.4. N Content and NUE under Different N-Supply Concentrations

**Figure 4:**
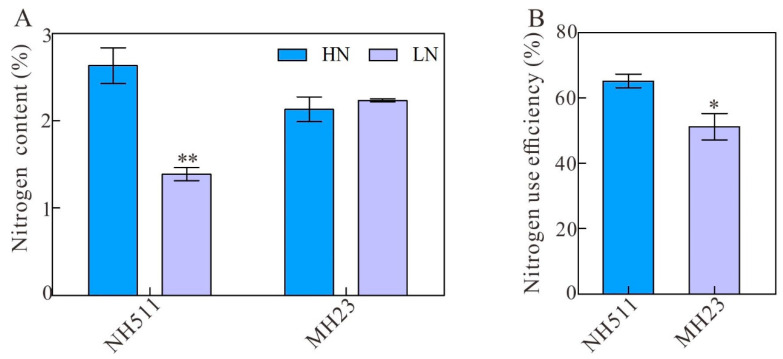

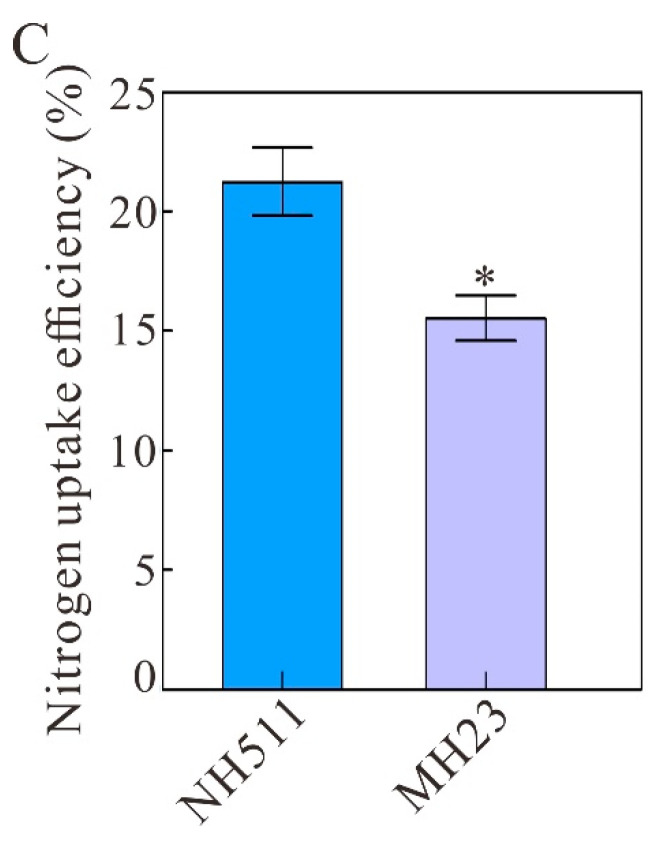
Measurement of nitrogen content and NUE under different N supplies. (**A**) Nitrogen content of NH511 and MH23 under HN and LN conditions. (**B**) Statistical analysis of NUE between NH511 and MH23 under HN supplies. (**C**) Analysis of N uptake efficiency between NH511 and MH23 under HN supplies. Statistical analysis comparing HN with LN was performed by *t*-tests in NH511 and MH23, respectively. **, *p* < 0.01; *, *p* < 0.05.

We concluded that under HN conditions, NH511 is an N-responsive cultivar, whereas MH23 is not. To investigate whether NH511 accumulates higher amounts of total N and exhibits higher NUE than MH23 when transferred into HN conditions, the total N content and NUE of seedlings of both cultivars were analyzed. The results showed that NH511 could accumulate more N than MH23 (Figure 4A), and significant differences were observed between these two cultivars, including NH511 showing a higher NUE than MH23 under the same conditions (Figure 4B). Similarly, the N uptake efficiency of NH511 is also higher than that of MH23 under the same conditions (Figure 4C). Overall, these results further demonstrated that NH511 is sensitive to different N levels and promotes its growth by increasing its N uptake and NUE and enhancing the activity of its metabolizing enzymes.

### 3.5. NH511 Improves Grain Yield by Increasing the Number of Tillers under High-N Supply

**Figure 5:**
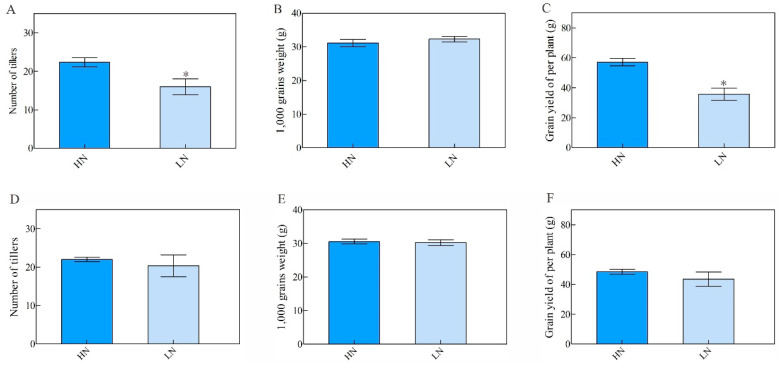
Agronomic traits of NH511 and MH23 in the field test. (**A**,**D**) Number of tillers was analyzed in NH511 and MH23, respectively, under HN and LN conditions. (**B**,**E**) 1000 grains weight analysis in NH511 and MH23, respectively, under HN and LN conditions. (**C**,**F**) Statistical analysis of grain yield per plant in NH511 and MH23, respectively. Statistical analysis of comparing HN with LN was performed by *t*-tests. *, *p* < 0.05.

To evaluate the performance of NH511 and MH23 in the field, we designed a field trial for a period of two years and collected data under HN and LN conditions. Our data showed that the grain yield of NH511 increased under HN conditions, as indicated by an increase in its tiller number (Figure 5A,C); however, its 1000-grain weight did not change under HN conditions (Figure 5B). Furthermore, our field test data indicated that NH511 should be used in rice breeding as it possesses the capacity for high production under high-N supply, similar to that of seedlings grown in a hydroponic solution. Notably, these data provide a reference basis for the selection of elite rice restorers and for high-NUE rice breeding.

### 3.6. Differential Transcriptome Signature Underlies the Variation in N Metabolism between NH511 and MH23

**Figure 6:**
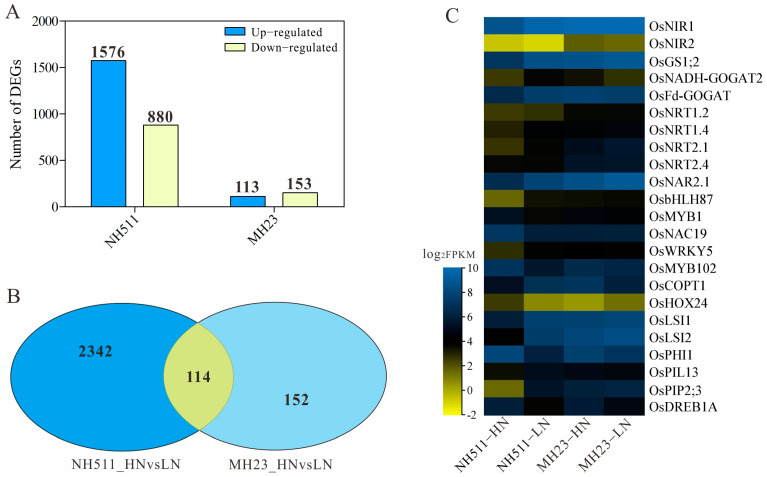
Comparative transcriptome analysis for NH511 and MH23 under HN supplies. (**A**) DEGs of NH511 and MH23 under HN supplies. (**B**) Number of the common differential genes in NH511 and MH23 under HN and LN supplies. (**C**) Heat map analysis of significant differentially expressed genes related to N and ion transportation. The heat map represents the relative expression levels based on FPKM values.

**Figure 7:**
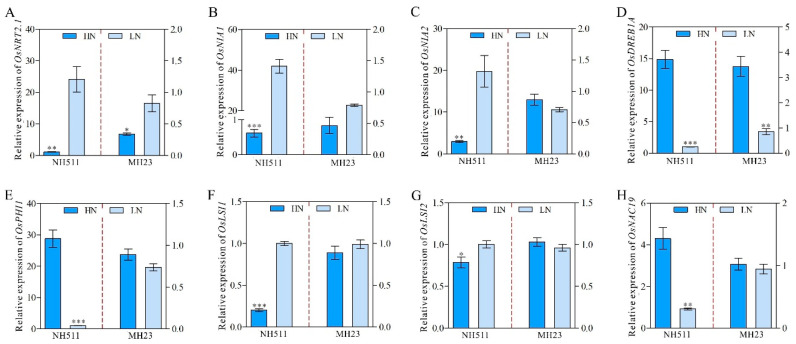
Real-time PCR validation of partial differential genes in NH511 and MH23. (**A**) Expression level of *OsNRT2.1* under HN and LN supplies. **(B**) Expression level of *OsNIA1*. (**C**) Expression level of *OsNIA2*. (**D**) Expression level of *OsDREB1A.* (**E**) Expression level of *OsPHI1*. (**F**) Expression level of *OsLSI1*. (**G**) Expression level of *OsLSI2*. (**H**) Expression level of *OsNAC19*. Statistical analysis comparing HN with LN was performed by *t*-tests in NH511 and MH23. ***, *p* < 0.001; **, *p* < 0.01; *, *p* < 0.05.

The observed difference in the performance of NH511 and MH23 under HN and LN conditions prompted us to further investigate the variations between the two cultivars. Based on transcriptomic data, differential expression analyses were performed using DESeq, which provided statistical routines for determining differential expression in digital gene expression data using a model based on a negative binomial distribution. The results showed that under HN conditions, 2456 genes were differentially expressed in NH511, including 1576 upregulated and 880 downregulated genes (Figure 6A and Figure S2A). Notably, under HN conditions, we identified only 266 differentially expressed genes (DEGs) in MH23, including 113 upregulated and 153 downregulated genes (Figure 6A and Figure S2B). Moreover, 114 DEGs were identified in both NH511 and MH23 under HN conditions (Figure 6B). Additionally, all DEGs were analyzed, and the known QTLs related to NUE were subsequently annotated (Figure S3). These DEGs are categorized into three groups: biological process (BP); cellular component (CC); and molecular function (MF) (Figure S4A,B). These DEG data showed that most of the DEGs were involved in metabolic activity and the stimulus response of BPs under HN conditions. Additionally, the heat map data revealed that under HN conditions, N-utilization genes and some transport-related genes, such as NRT1.2, NRT1.4, NIA1, NIA2, and NAC19, exhibited evident differential expression in NH511 but not in MH23 (Figure 6C). These DEGs were further confirmed by real-time PCR, and the results showed significant differential expression variations in NH511 under N supplies (Figure 7). Overall, the transcriptome data provided additional insights into the contrasting responses of NH511 and MH23 to varying N supplies, particularly in terms of their differential gene expression. These findings offer potential avenues for identifying key NH511 genes that are involved in N response and contribute to high NUE in future rice-breeding endeavors.

## 4. Discussion

N is an essential mineral nutrient required for plant growth and development, particularly in crops [35,36]. N deficiency triggers extensive physiological and biochemical changes in plants, and a low N-utilization rate has become one of the main abiotic factors affecting crop growth [21,37]. Excess N is inevitably leached into the underground water system and lost to the atmosphere, resulting in severe environmental problems [28]. A previous study has revealed that soil acidification in China is primarily a consequence of high-N fertilizer inputs [38]. Nevertheless, reducing the use of N fertilizers and cultivating high-NUE cultivars are efficient ways to solve environmental problems and ensure adequate crop production.

In China, the breeding of semi-dwarf rice varieties led to an increase in rice yield from 2.0 t ha^−2^ in the 1960s to 3.5 t ha^−2^ in the 1970s [4]. China has been successful in breeding hybrid rice in the past decades but is now facing challenges in developing new hybrids with high-yielding potential, better grain quality, and tolerance to biotic and abiotic stresses [4,39,40,41,42]. Hybrid rice has been successfully developed with a three-line system based on a sterile male line, a maintainer line, and a restorer line, which greatly contributed to global food production in the past decades [6,42,43]. NH511 was derived from the hybridization between two heavy panicle-type restorer lines, namely, Shuhui 881 and Shuhui 527, which exhibited good combining ability and acceptable grain quality [44]. NH511 has been widely used in hybrid seed production as a restorer line and has been used to breed some hybrid rice cultivars in the past. MH23 was derived from the hybridization between two restorer lines, namely, IRBB23 and Minghui 63, the latter being a well-known indica rice restorer line that has been widely applied to hybrid rice seed production in China [45,46].

The results of our previous study revealed that different rice cultivars originating from all over China exhibited diversity in N utilization based on a comprehensive analysis of large-scale screening and N-supply data [31]. In the present study, we selected two specific indica restorer lines, NH511 and MH123, to further investigate this topic and provide novel insights into the N utilization of rice restorer lines and high-NUE rice breeding. Our analysis of morphological variations and N-metabolizing enzymes of NH511 and MH23 grown under HN and LN conditions showed that NH511 was an N-responsive restorer line whose seedling growth was promoted by the regulation of lateral root development and plant height (Figure 1 and Figure 3). The chlorate assays and NUE analysis results showed that compared with MH23, NH511 had a high nitrate absorption rate at the seedling stage and showed excellent performance in field tests (Figure 2, Figure 4, and Figure 5). Finally, transcriptome analysis demonstrated a variation signature between NH511 and MH23 in response to different N-supply concentrations, and differential genes were further confirmed by real-time PCR (Figure 7).

Overall, our results revealed physiological and gene-expression variations between the two restorer lines, showing that NH511 is an excellent rice restorer line owing to its high NUE, good combining ability, and acceptable grain quality. Further investigation is needed to determine the specific genes responsible for the increased number and length of lateral roots in NH511 under HN conditions, elucidating the underlying molecular mechanism. Additionally, the mechanism underlying the high NUE in NH511 can be elucidated more specifically using other advanced biotechnologies, particularly those related to gene identification, as well as by further functional analyses. Finally, the high-NUE genes identified in NH511 could be used for rice breeding, which would not only help resolve certain environmental issues but also ensure crop production.

## 5. Conclusions

We investigated the physiological and transcriptomic changes in two indica restorer lines (NH511 and MH23) under HN and LN conditions. Based on our findings, we discussed and compared the N-responsive characteristics of the two cultivars and their DEGs under HN conditions. Compared to MH23, NH511 accumulated more N and had higher NUE under the same conditions. We described the physiological mechanisms underlying these differences (namely, the regulation of lateral development and plant height) and concluded that NH511 was an excellent rice restorer line. Our study provides novel insights into rice breeding because we described the regulatory activities involved in the N response in two rice restorer lines, indicating their further uses and perspectives for future studies.

## Data Availability

Not applicable.

## Supplementary Materials

The following supporting information can be downloaded at: https://www.mdpi.com/article/10.3390/plants12122276/s1, Figure S1. Content of soluble proteins under HN and LN conditions. Figure S2. Volcano plot of DEGs identified under HN supplies. Figure S3. Annotation of the known QTLs related to NUE based on DEGs under different N supplies. Figure S4. Histogram description of Gene Ontology enrichment of DEGs. Table S1. qRT-PCR primers used in this study.
