# Supplementary material for: Transcriptomic and Physiological Analyses of Two Rice Restorer Lines under Different Nitrogen Supplies Provide Novel Insights into Hybrid Rice Breeding
Source: Plants (Basel). 2023 Jun 11;12(12):2276. doi: 10.3390/plants12122276 (PMC10304098; doi:10.3390/plants12122276)
Supplement: Supplementary file 1 [file plants-12-02276-s001.zip › plants-2416433-supplementary.pdf]

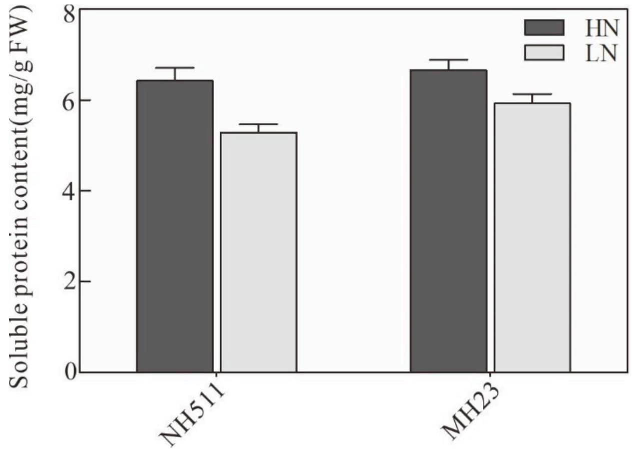

Figure. S1 Measurement of soluble proteins under different N supplies in NH511 and MH23 respectively. The statistical analysis of comparing HN with LN was performed by t-tests in NH511 and MH23 respectively.

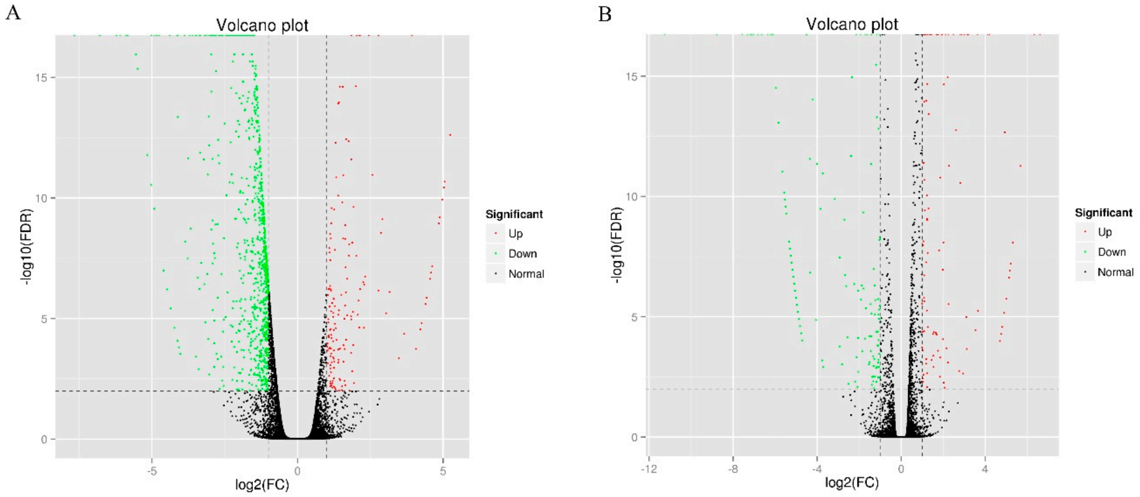

Figure. S2 Volcano plot of DEGs identified under different N supplies. A, DEGs of NH511. The red pots represent 1576 significantly up-regulated unigenes. The green pots represent significantly down-regulated 880 unigenes. The black pots mean no significantly differential expression. B, DEGs of MH23 under HN and LN conditions. 113 significantly up-regulated unigenes and 153 down-regulated unigenes.

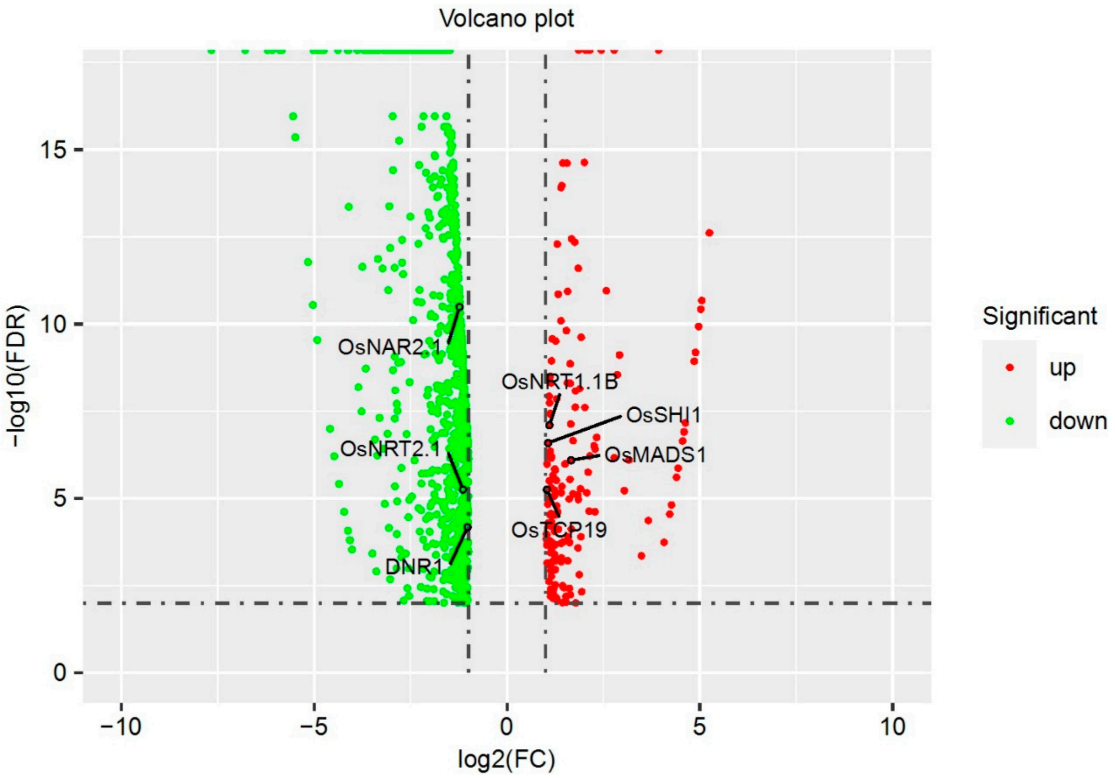

Figure. S3 Annotation of the known QTLs related to NUE based on DEGs under different N supplies. All DEGs were analyzed and the known QTLs related to NUE, including *OsTCP19*, *OsMADS1*, *OsSHI1*, *OsNRT1.1B*, *OsNAR2.1*, *OsNRT2.1* and *DNR1*, were identified and annotated subsequently.

A

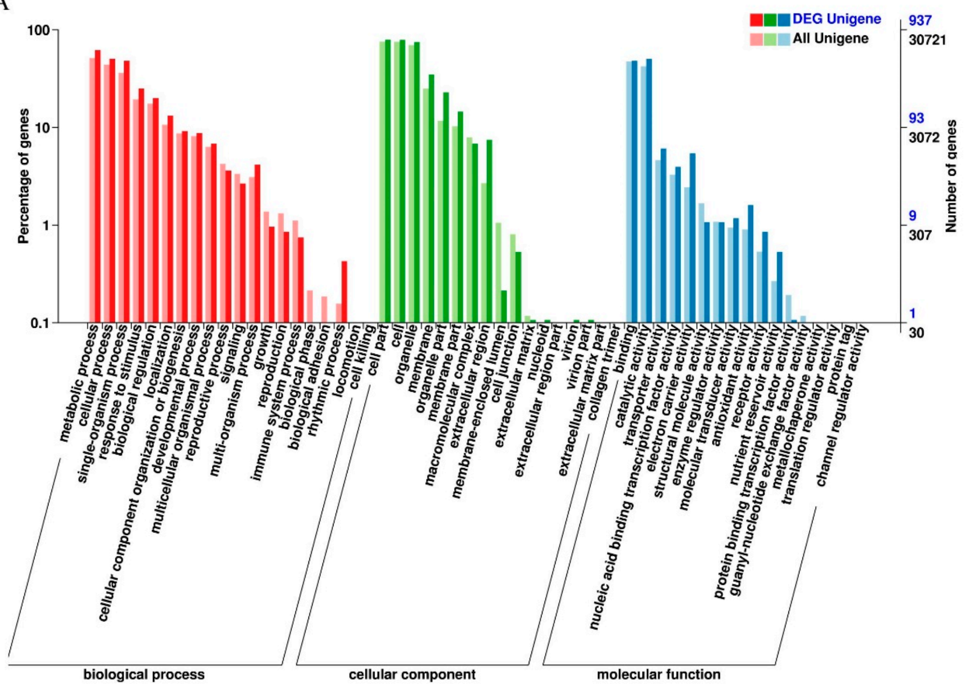

B

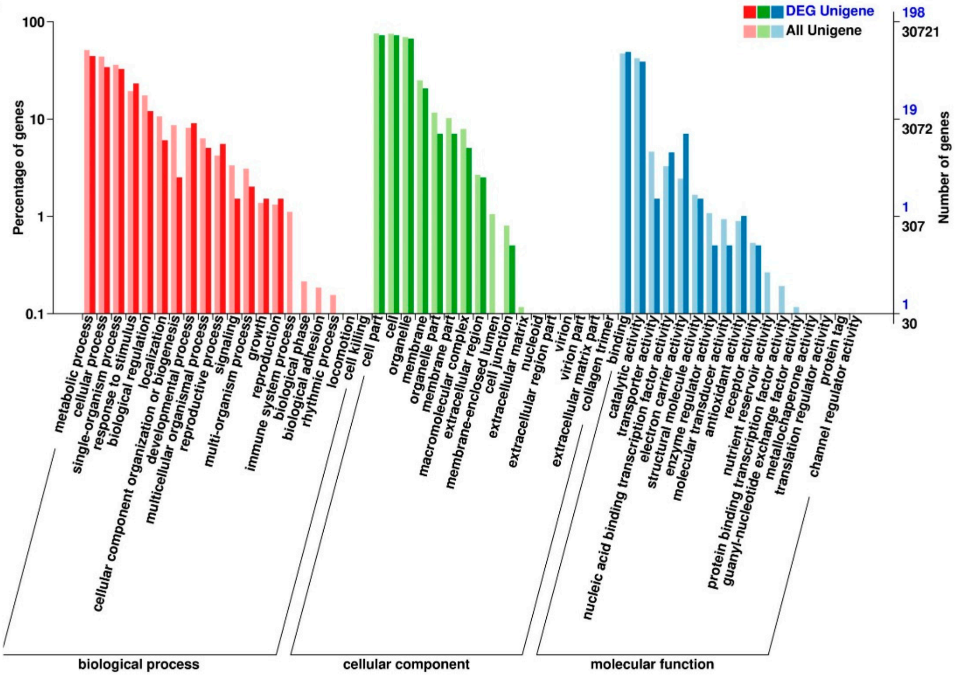

Figure. S4 Histogram description of Gene Ontology enrichment of DEGs. A, GO analysis of DEGs in NH511. B, GO analysis of DEGs in MH23. The DEGs were fell into three categories: biological process (BP), cellular component (CC) and molecular function (MF). The X-axis represents various gene function, the Y-axis corresponds to the number of DEGs.

Table S1. qRT-PCR primers used in this study.

Primer name	Sequence(5'-3')
ACTIN1-F	ACCATTGGTGCTGAGCGTTT
ACTIN1-R	CGCAGCTTCCATTCCTATGAA
OsNIA1-F	TCAAGGTGTGGTACGTGGTG
OsNIA1-R	CGAGGTCATAGCCCATCTTC
OsNIA2-F	TGTACCAGGTCATCCAGTCG
OsNIA2-R	CGATGACGTACCACACCTTG
OsNRT2.1-F	CTTCACGTCGTCGAGGTACT
OsNRT2.1-R	CACTCGGAGCCGTAGTAGTG
OsPHI1-F	TCGACGGCTTCTGCATGAG
OsPHI1-R	GATCATCGAGGCGAGGGATATC
OsLSI1-F	CCATAGCGATACAGTACAGGAG
OsLSI1-R	GACGAACATCATGTTGAACGTC
OsLSI2-F	CTGGAGATGTCGGAGAACATAA
OsLSI2-R	GAAGCTCTTGAGGAACAGCTTC
OsDREB1A-F	GAACTGGACGTCCTGAGTG
OsDREB1A-R	CTAGTAGCTCCAGAGTGGGA
OsNAC19-F	GAAGAACGAGTGGGAGAAGATG
OsNAC19-R	TCGTCCATCGATTCTTTCTTGG
